# Prevalence of hepatitis C virus in mothers and their children in Malawi

**DOI:** 10.1111/tmi.12465

**Published:** 2015-02-06

**Authors:** James M Fox, Robert Newton, Marija Bedaj, Ada Keding, Elizabeth Molyneux, Lucy M Carpenter, Fabiola Martin, Nora Mutalima

**Affiliations:** 1 Centre for Immunology and Infection, University of YorkYork, UK; 2 Epidemiology and Cancer Statistics Group, University of YorkYork, UK; 3 MRC/UVRI Uganda Research Unit on AIDSEntebbe, Uganda; 4 York Trials Unit, University of YorkYork, UK; 5 Department of Paediatrics, College of MedicineBlantyre, Malawi; 6 Nuffield College, University of OxfordOxford, UK

**Keywords:** Hepatitis C, hepatitis C virus, Malawi, prevalence, serology, epidemiology

## Abstract

**Objectives:**

Hepatitis C virus (HCV) prevalence is poorly mapped in the East African region; with the advent of novel HCV therapies, better epidemiological data are required to target the infection. We sought to estimate HCV prevalence in healthy Malawian mothers and assess mother-to-child transmission (MTCT); context is provided by reviewing previously published HCV prevalence data from the region.

**Methods:**

Using ELISA screening and confirmatory blot, serological testing of 418 healthy Malawian mothers for HCV was performed. To examine MTCT, the children of any positive women were also tested for HCV; all children had malignant disease unrelated to hepatocellular carcinoma. We compared our results to published literature on HCV prevalence in Malawi and its neighbouring countries.

**Results:**

Three of 418 women were HCV reactive by ELISA; two were confirmed positive by immunoblot (0.5%). One child of an HCV-infected mother was HCV seropositive. The literature review revealed HCV prevalence ranging from 0 to 7.2% in the region, being highest in Tanzania and specifically for cohorts of inpatients and HIV-co-infected people. The overall estimated prevalence of HCV in Malawi was 1.0% (95%CI 0.7–1.4) when all studies were included (including this one), but lower in healthy cohorts alone at 0.3% (95%CI 0.1–1.2).

**Conclusions:**

This is the first study using confirmatory tests to examine HCV prevalence in healthy Malawian mothers; the prevalence was low. Future studies need to address the source of infection in healthy women.

## Introduction

Hepatitis C virus causes hepatitis, cirrhosis and hepatocellular carcinoma [1]. It is a bloodborne infection and prevalent among intravenous drug users (IVDU) [2] and men who have sex with men (MSM), particularly those with HIV co-infection [3,4] and their contacts. African and Asian countries are thought to have the highest burden of infection, but current prevalence estimates are variable [5]. In Malawi, the reported prevalence of HCV in healthy blood donors ranges from 0.1% to 18% [6,7]. Heterosexual transmission has been reported as an unlikely route of transmission in Malawi [8]. Other modes of transmission, such as iatrogenic spread in medical facilities, or among intravenous drug users, are thought to be rare [9,10]. A study from Tanzania, a neighbouring country, reports that MTCT is low [11], but this has not been assessed in Malawi.

We examined the prevalence of HCV retrospectively as part of an ongoing study of infections and cancers among children in Malawi [12]. We report our findings in the context of previous HCV prevalence studies from Malawi and its neighbouring countries by way of a comprehensive literature review. Mapping HCV prevalence is a prelude to possible eradication of HCV in lower income countries [13] following recent success with novel HCV therapies [14].

## Materials and methods

Sera from 418 paired Malawian women and their children were collected between 2006 and 2010 and stored at −80 °C until testing. Samples had been originally collected as part of a childhood malignancy and bloodborne virus (BBV) study at the Queen Elizabeth Central Hospital in Blantyre, Malawi [12]. The majority of children had been diagnosed with Burkitt's lymphoma and the remainder had a range of other tumours. Ethical approval for the study was obtained from the Oxford Tropical Research Ethics Committee and the Malawian College of Medicine Research and Ethics Committee. Details of the original study are published elsewhere [12].

Sera from the healthy women were screened in duplicate by MP Diagnostics HCV ELISA 3.0 following manufacturer's instructions (MP Biomedicals, Cambridge, UK). Borderline and reactive samples were retested. Repeatedly ELISA reactive sera underwent confirmatory testing using HCV BLOT 3.0 (MP Biomedicals). Positivity was defined according to the manufacturer's guidelines. Sera of children of HCV-positive mothers were tested using both ELISA and immunoblot. The ELISA used in this study tests for the detection of antibodies in sera towards the HCV antigens NS5, NS3 (c 200), NS4 (c 200) and the core (c22); the strip immunoblots are a qualitative enzyme immunoassay and contain recombinant HCV proteins from the capsid, NS3, NS4 and NS5 regions of the HCV genome; higher sensitivity is reported for the immunoblots compared to the ELISA.

For the comprehensive review, PubMed was searched in March 2014 using methods appropriate for systematic review; the search terms were as follows: ‘HCV’ or ‘Hepatitis C’ in combination with ‘Malawi’, ‘Mozambique’, ‘Tanzania’ or ‘Zambia’. Data of demographic factors, cohort size, HCV prevalence and whether or not a confirmatory test (CT) was employed were extracted. Publications were excluded that only reported reactive screening tests in the absence of CT, HCV prevalence in refugee cohorts and in patients with jaundice or liver disease. Total HCV prevalence by country, standard error and 95% confidence interval, using the Wilson interval to take account of the small anticipated prevalence, were calculated where more than one study was available.

Informed and written consent was obtained from all participants according to Oxford Tropical Research Ethics Committee and the Malawian College of Medicine Research and Ethics Committee ethics.

## Results

### HCV screening

Paired sera from 418 paired Malawian women and children were available for testing. All children attended the hospital for childhood malignancies; none had hepatocellular carcinoma. At the time of sampling, the children were of a mean age of 6.6 years. Three of 418 mothers' sera were repeatedly HCV ELISA reactive. Two reactive samples were confirmed to be positive by immunoblot. All three children of these mothers were ELISA non-reactive, but the serum of one of these children was repeatedly HCV positive in the more sensitive immunoblot test. We therefore report two (0.5%) HCV-positive mothers and one HCV-positive child. The 51-year-old HCV-positive woman with a HCV-negative child was HIV negative and reported one lifetime sexual partner. The second positive mother was also HIV negative and reported one lifetime sexual partner; her HCV-positive daughter was 4 years of age, HIV negative and had no blood transfusion history. Fresh whole blood was unavailable; therefore, HCV RNA levels could not be measured to report disease activity.

### Comprehensive review

To increase our understanding of the HCV prevalence in the East African region around Malawi, we conducted a comprehensive review of published HCV prevalence data. Our search returned a total of 50 studies, of which 14 met the inclusion criteria. Of those, four publications were from Malawi, three from Mozambique, six from Tanzania and one from Zambia (Table 1). The HCV prevalence ranged from 0% to 7.2% and was highest regionally in Tanzania and for patient cohorts of hospital inpatients and HIV-co-infected people. The prevalence rates aggregated by country are summarised in Table 2. The overall estimated prevalence of HCV in Malawi was 1.0% (95%CI 0.7–1.4) when all studies were included, including the one reported here, but was much lower in healthy cohorts alone at 0.3% (95%CI 0.1–1.2). The HCV prevalence in Tanzania appeared higher (2.1% [95%CI 1.6–2.6]), but this might be due to high prevalence of HCV in just one study among apparently healthy women [15]. In our analysis, Mozambique had an HCV prevalence intermediate between Malawi and Tanzania; Zambia's prevalence was similar to healthy Malawians, but only one study met our inclusion criteria (Table 2).

**Table 1:** Published prevalence of HCV in Malawi and its neighbouring countries

First author/publication year	Nation	Cohort (*n*)	Healthy	Non-healthy	Sex	HCV prevalence (%)
Chimphambano *et al*. [20]	Malawi	142	HV		M	0.0
		22	HV		F	0.0
Nyirenda *et al*. [9]	Malawi	77		IP	M	2.6
		125		IP	F	5.6
Moore *et al*. [16]	Malawi	299		HIV+	ND	5.7
Chasela *et al*. [7]	Malawi	2040		HIV+ PW	F	0.1
Dazza *et al*. [24]	Mozambique	194	BD		ND	2.1
Cunha *et al*. [25]	Mozambique	1231	BD		M	1.4
		346	BD		F	1.8
Stokx *et al*. [26]	Mozambique	609	BD		M	0.0
		70	BD		F	0.0
Miller *et al*. [27]	Tanzania	212	HV		M	0.0
Menendez *et al*. [11]	Tanzania	980	PW		F	2.4
Tess *et al*. [28]	Tanzania	221	HV		M	1.8
		295	HV		F	0.7
Stark *et al*. [15]	Tanzania	208	HV		F	7.2
Msuya *et al*. [29]	Tanzania	382	HV		F	1.1
Puato *et al*. [30]	Tanzania	750	HV		ND	2.0
Oshitani *et al*. [31]	Zambia	240	BD		ND	0.0
		343		IP	ND	0.3

F, female; M, male; ND, not defined; PW, pregnant women; IP, inpatient; BD, blood donors; HV, healthy volunteer; HIV+, HIV positive.

**Table 2:** Estimated prevalence of HCV in Malawi and its neighbouring countries

Country	Number of studies	Cohort (*n*)	HCV prevalence (%)	SE	95% CI
Malawi (all)	5	3123	1.0	0.17	0.7–1.4
Malawi (healthy)	2	582	0.3	0.24	0.1–1.2
Mozambique (healthy)	3	2450	1.1	0.21	0.8–1.6
Tanzania (healthy)	5	3048	2.1	0.26	1.6–2.6

SE, standard error; CI, confidence interval. ‘Healthy’ is defined as male/female, non-inpatient and not declared HIV positive.

## Discussion

This seroprevalence study of HCV in paired mothers and their children in Malawi detected a low HCV prevalence of 0.5% in a cohort of healthy women who represent the female general population of Malawi. Our review showed that HCV prevalence is low in healthy adult volunteers and blood donors in the East African region, but higher among individuals who were hospitalised or HIV co-infected [16,9]. Tanzania has the highest and Zambia the lowest reported HCV prevalence. Our literature review purposefully concentrated upon Malawi and its neighbouring countries and only included studies that used comparable methodological stringency (screening + confirmation); other reviews on HCV prevalence in Africa, which include some data for these East African nations, are available for HIV-co-infected individuals [5,17], pregnant women [18] and general populations [19].

Although we confirmed low prevalence of HCV in healthy women, one of two children of HCV-positive mothers was HCV positive. A study from Tanzania reported no MTCT in 43 children born to HCV-positive mothers [11].

The exact transmission risk factors for our cohort remain uncertain. In general, contaminated-needle re-usage or contaminated-needle-stick injuries, contaminated blood transfusions and unprotected sexual intercourse, especially in HIV-positive men, are highly associated with HCV transmission.

Blood transfusions are routinely tested for HCV in Malawi, but we are not aware of needle exchange programmes for harm reduction. HCV prevalence in IVDU and HIV-positive MSM has not been studied extensively in this region. Both factors may directly or indirectly play a role in HCV transmission to women. A study by Chimphambano *et al*. [20] examined HCV prevalence in male and female Malawian prison inmates. While 86% of the inmates reported being aware of intercourse between male prisoners and 2.1% identified themselves as being homosexual, HCV prevalence was reported to be 0%. Injectable drug use was not reported in this study. Only one study in Tanzania [21] has compared HCV prevalence in this high-risk group, examining HCV prevalence in 267 injecting drug users (IDU) and 163 drug users that had never injected (NIDU). The IDUs had a HCV prevalence of 27.7% whereas that of NIDUs was 1.8%, although no confirmatory test was performed. A further study [22] did not meet our inclusion criteria but conducted research in 509 Tanzanian MSM and found HCV prevalence to be 14%. IVDU is rare in women compared to men, but data in our cohort for IVDU or having contact/living with someone who was an active intravenous drug user were not available to us and neither was the transfusion history and possibility of iatrogenic spread for the mothers. The HCV-positive child had no recorded blood transfusions, but information on the child being breastfed by a wet nurse instead of its mother or any other medical intervention was similarly not available.

Malawi is known to have a high HIV prevalence [23], and HIV infection is a known risk factor for HCV infection. However, neither of the HCV-infected women in our study were HIV co-infected. HIV prevalence in paediatric inpatients in Malawi has been reported to be 8.8% [23], but the HCV-positive child in our cohort was also HIV negative.

Therefore, we can only hypothesise that the mothers may have acquired their HCV from having been either breastfed as a child by somebody infected or from contact with an infected partner or contaminated needles/instruments or transfusions before transfusion screening for bloodborne viruses was in place. The HCV-positive child may have been infected through contact with contaminated needles/instruments or by being breastfed by a HCV-positive wet nurse.

Our study has several strengths: the sample size studied was large, children's sera were matched, and both ELISA screening and a more sensitive confirmatory test were employed. We compared our data with HCV prevalence of neighbouring countries by conducting a literature review. The study's limitation was the relative age of sera, the fact that not all children were tested for HCV irrespective of their mother's results and that fresh whole blood samples were not available to test for HCV RNA and liver function.

In summary, we report that HCV is prevalent at low levels in mothers living in Malawi. It is unclear how these women acquired their infection. HCV high-risk groups have not been studied in this region. Research on these groups and their partners and children may inform transmission prevention and eradication programmes.
